# Effects of Pre-Deformation in Corrosion Fatigue Crack Growth of Al-Mg-Zn Alloy

**DOI:** 10.3390/ma18020365

**Published:** 2025-01-15

**Authors:** Hui Jiang, Junjun Jin, Yu Fang, Guoqing Gou, Wei Lu, Zhiyi Zhang, Hongmei Zhou, Hairong Sun, Jikui Feng, Jia Chen, Zhenghong Fu

**Affiliations:** 1 School of Mechanical Engineering, Chengdu Industry and Trade College, Chengdu 610031, China; jianghui@cdgmxy.edu.cn (H.J.); fangyu@cdgmxy.edu.cn (Y.F.); 2 State Key Laboratory of Precision Manufacturing for Extreme Service Performance, Central South University, Changsha 410083, China; jinjun2358@163.com (J.J.); fuzhenghong@swjtu.edu.cn (Z.F.); 3 Key Laboratory of Advanced Technologies of Materials, Ministry of Education, School of Materials Science and Engineering, Southwest Jiaotong University, Chengdu 610031, China; swjtuluwei@163.com; 4 CRRC Qingdao Sifang Co., Ltd., Qingdao 266111, China; zhangzhiyi@cqsf.com; 5 School of Materials and Environmental Engineering, Chengdu Technological University, Chengdu 610031, China; angriy@163.com; 6 Sichuan Special Equipment Inspection Institute, Chengdu 610031, China; shr-19@163.com; 7 Hubei Aerospace Flight Vehicle Institute, Wuhan 430000, China; 18327030324@139.com; 8 Applied Mechanics and Structure Safety Key Laboratory of Sichuan Province, School of Mechanics and Aerospace Engineering, Southwest Jiaotong University, Chengdu 610031, China

**Keywords:** Al-Mg-Zn alloy, pre-deformation, corrosion fatigue, cracking path, dislocation density

## Abstract

This study investigated the effect of pre-deformation on the corrosion fatigue crack propagation (CFCG) of Al-Mg-Zn alloy in a corrosive environment. Tensile tests at different pre-deformation levels and molecular dynamics simulations analyzed changes in dislocation density. Corrosion fatigue experiments were conducted in a 3.5% NaCl solution at room temperature, and crack propagation morphology was characterized using electron backscatter diffraction (EBSD), scanning electron microscopy (SEM), and transmission electron microscopy (TEM). The results showed that tensile strength increased by 2.63% and 10.00% for 5% and 10% pre-deformation, respectively. The crack propagation threshold values were L_2_ (6.36 MPa·m^1/2^) > L_0_ (6.05 MPa·m^1/2^) > L_1_ (5.13 MPa·m^1/2^), attributed to increased dislocation density and material strength. At 5% pre-deformation, dislocation pile-ups created stress concentrations that facilitated crack propagation. In contrast, the non-uniform dislocation distribution at 10% pre-deformation enhanced both material strength and resistance to crack growth.

## 1. Introduction

Al-Mg-Zn alloy is widely used in high-speed trains for its high strength-to-density ratio and good fatigue resistance. It is 35% lighter than traditional materials like stainless steel and weathering steel, allowing for increased load capacity and reduced energy consumption under the same traction conditions [1,2,3]. A dense oxide layer is readily formed on the surface of the alloy, which prevents oxidative corrosion. Youhong Peng et al. revealed the characteristics of sustained load crack growth in Al-Zn-Mg-Cu aluminum alloys in a saline environment [4]. Melika Jalali et al. studied how ARB (Accumulated Roll Bonding) as a severe plastic deformation technique alters the microstructure and corrosion resistance of this alloy. The results indicated that, due to the higher dislocation density, an increased number of ARB cycles initially leads to a decrease in corrosion resistance [5]. Fatigue refers to the fracture phenomenon caused by an accumulation of pre-deformation in metals and their structural parts under the action of long-term loads. The damage caused by fatigue fracture accounts for more than 80% of total operation failures. Lingjian Meng et al. found that flaky α fracture releases a greater local stress concentration during low-temperature pre-stretching, thereby maintaining the high work hardening capacity of the alloy [6]. The corrosion fatigue of metallic materials is a form of corrosion–plastic failure under the influence of alternating loads and corrosive environments. This involves a process of mutual promotion of corrosion and fatigue damage [7,8,9,10,11]. The operating conditions of high-speed trains necessitate exposure to complex and diverse conditions, such as humidity, marine atmospheres, and industrial pollution environments. Corrosive media such as Cl^−^ and NO_3_^−^ in a humid environment will affect aluminum alloy materials and aluminum alloys, notably welded joints, which can experience corrosion damage. In addition, the aluminum alloy body of high-speed trains and the associated welded structural parts are subject to a constant load and alternating cyclic loads over extended periods. Therefore, the service reliability of the aluminum alloy body and the welded structural parts is a critical guarantee for the safe operation of high-speed trains [12,13,14].

During actual operation, the cover plate made of Al-Mg-Zn alloy in key components of high-speed trains is responsible for withstanding both the cyclic load and the dead weight of the corbel. Consequently, the Al-Mg-Zn Alloy cover plate will exhibit a certain degree of pre-deformation during operation and service. Pre-deformation has a significant impact on the crack propagation behavior of aluminum alloys. Through pre-deformation, the grain structure of aluminum alloys can be refined, and the degree of bending and twisting of grain boundaries increases, thereby enhancing the material’s strength and toughness and inhibiting crack propagation. At the same time, pre-deformation also alters the residual stress distribution of aluminum alloys. If pre-deformation introduces stress concentration areas, it may accelerate crack propagation. Additionally, pre-deformation increases the dislocation density of aluminum alloys. A moderate dislocation density can enhance the material’s strength and toughness [15], suppressing crack propagation, but excessive dislocation density may lead to easier crack propagation. Therefore, in the design and application of aluminum alloy materials, it is necessary to comprehensively consider the influence of pre-deformation on crack propagation behavior to improve the material’s resistance to crack propagation, However, there is still insufficient research on the fatigue behavior and corrosion effects of aluminum–magnesium–zinc alloys under pre-deformation conditions, especially regarding the long-term durability of aluminum alloys in high-humidity environments and marine climates, where relevant literature is scarce. Therefore, this study aims to fill this research gap by systematically analyzing the impact of pre-deformation on the fatigue and corrosion performance of aluminum alloys, providing a theoretical foundation for the optimization design and application of aluminum alloy materials in future high-speed trains.

This study aims to investigate the corrosion fatigue crack propagation (CFCG) behavior of Al-Mg-Zn alloy aluminum alloys under different levels of pre-deformation. Microscopic analysis of the aluminum alloy’s microstructure, residual stress, crack orientation, and fracture morphology was conducted using optical microscopy, electron backscatter diffraction (EBSD), scanning electron microscopy (SEM), and transmission electron microscopy (TEM). Furthermore, molecular dynamics simulation methods were employed to explore the changes in dislocation density resulting from different levels of pre-deformation, which to some extent explains the differences in corrosion fatigue crack propagation rates and mechanisms under different plastic damage conditions.

## 2. Materials and Methods

### 2.1. Materials and Mechanical Properties

**Figure 1:**
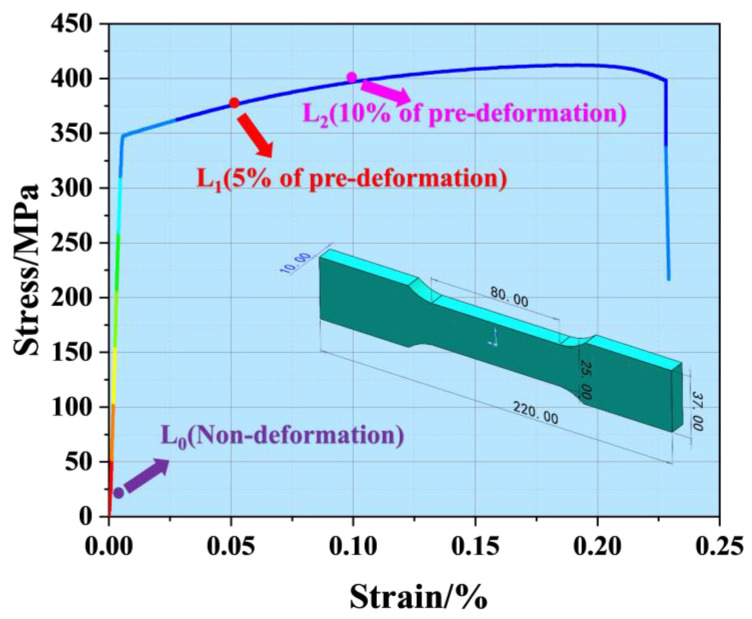
Stress–strain curve of Al-Mg-Zn Alloy base metal.

In this study, Al-Mg-Zn alloy was used as the research object. The composition is given in Table 1. The Al-Mg-Zn Alloy sheet was processed into standard tensile specimens by wire cutting, according to the standard tensile specimen size in Figure 1. The sample thickness was 10 mm.

The stress–strain curve for the Al-Mg-Zn alloy is illustrated in Figure 1. In this study, we prepared three distinct groups of samples: Group L_0_ (non-deformation), Group L_1_ (5% pre-deformation), and Group L_2_ (10% pre-deformation). Each sample was machined to standardized dimensions and mounted on a DNS300 tensile testing machine (Changchun Machinery Research Institute, Changchun, China). During the pre-deformation process, tensile loads were applied to the L_1_ and L_2_ samples until strains of 5% and 10% were achieved, respectively. An extensometer continuously monitored the strain, and each sample was held under the applied load for 10 min to ensure consistent sample deformation. After this period, the samples were unloaded and prepared for further mechanical testing to evaluate the effects of pre-deformation on their stress–strain behavior.

**Figure 2:**
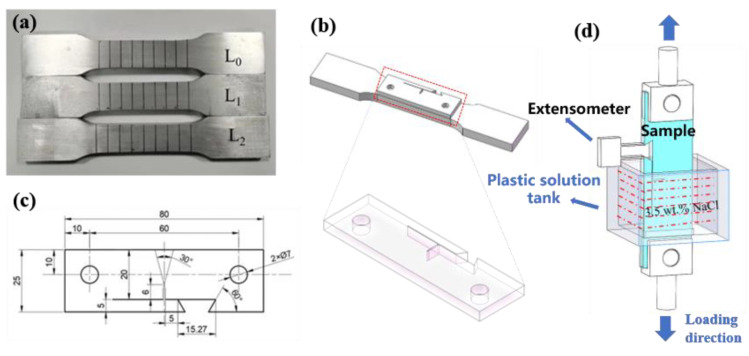
Pre-deformation of CFCG: (**a**) tensile specimen with different degrees of pre-deformation; (**b**) schematic diagram of the sampling position of the crack growth sample; (**c**) the size of the crack growth sample; and (**d**) schematic diagram of the crack growth device.

Figure 2a and b illustrate the improved unilateral notch specimen (improved Single edged notched tension, (SENT) specimen) and the sampling position of the CFCG. The specimen was provided with an extensometer slot above the notch to facilitate a fixed extensometer. The size of the improved SENT sample is shown in Figure 2c, and the crack expansion device is given in Figure 2d. Samples were taken from the three groups: L_0_, L_1_, and L_2_. The crack length data were collected in real time using a computer program interfaced with the extensometer. The computer program was written in Visual Basic (VB) language, the electronic extender adopted model Y5/2.5 for data acquisition, the extender range was 5 mm, and the measuring range was 2.5 mm.

### 2.2. Microstructure

The metallographic samples were treated with sandpaper of different grit sizes (180–2000), and then polished on a polishing machine until there were no detectable surface scratches. After polishing, the samples were cleaned with alcohol and then treated with acid (2% HF, 3% HCL, 5% HNO_3_, and 90% H_2_O). An optical microscope (OM) (model of equipment: Zeiss−A1M, Carl Zeiss AG, Oberkochen, Germany,) and scanning electron microscope (SEM) ((model of equipment: JSM−6490LV, JEOL, Tokyo, Japan) were used to observe the fracture of the corrosion fatigue crack growth sample. The element types and product components were then analyzed. Using electron back-scattered diffraction (EBSD) (model of equipment: NordlysNano, Oxford Instruments, Oxford, UK), the distribution of secondary phases and grain boundaries was investigated analytically. All samples were then electropolished in an electrolyte consisting of 10% roach acid and 90% ethanol. The electropolishing process was run at 25 V for a polishing time of 40 s. The distribution of dislocations and strengthening phases under different pre-deformations was investigated using an FEI Tecnai 20 TEM (model of equipment: Thermo Fisher Scientific, Hillsboro, OR, USA).

### 2.3. Molecular Dynamics Simulation

In order to probe possible microstructural changes under different degrees of pre-deformation, the molecular dynamics simulation software package LAMMPS (Large-scale Atomic/Molecular Massively Parallel Simulator: LAMMPS 3 Mar 2020) [16] developed by Sandia National Laboratory was used for simulation calculation. The data processing software used was the Open Visualization Tool (Ovito-3.11.3—OVITO), developed by Alexander Stukowski of Darmstadt University of Technology [17]. This visualization and analysis software for atomic and particle simulation data enables valuable insight into material phenomena and physical processes. The Voronoi algorithm and MEAM [18] (Modified Embed Atom Method) were employed to establish and calculate the tensile test of the polycrystalline model of Mg-Al alloy.

**Figure 3:**
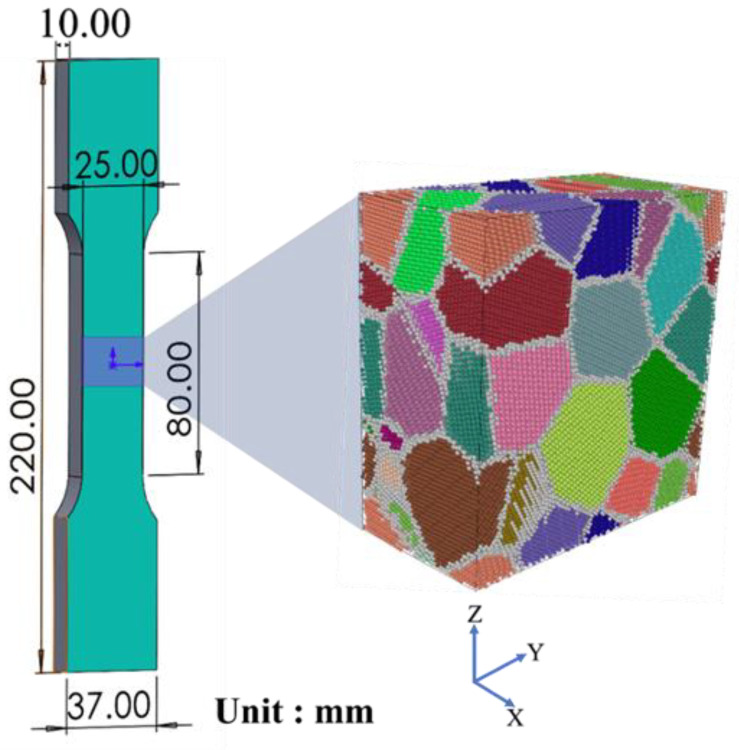
Molecular dynamic tensile model of Al-Zn-Mg alloy.

In order to facilitate modeling and calculations, we have neglected the influence of other trace elements. Figure 3 shows the simplified molecular dynamics tensile model of the Al-Mg-Zn alloy. The model represents a polycrystalline Al-Mg-Zn aluminum alloy of constant size (200 nm × 100 nm × 200 nm), where the lattice constant of Al atoms is “a = 4.05”. The model contains 240,877 atoms and 20 grains. The composition of the alloy is Al (wt%): Zn (wt%): Mg (wt%) = 94%: 4.6%: 1.4%. The X, Y, and Z coordinates correspond to the [100], [010], and [001] crystallographic directions.

Following model establishment and relaxation, a tensile load in the [0 0 1] direction was applied to the alloy system to assess the motion of atoms and the relationship between stress and strain during the entire stretching process. The temperature of the system was set at 300 K, and the X, Y, and Z directions are all periodic boundary conditions. A time step of 0.001 ps was used for the simulation, a strain rate of 0.001 was applied, and the system was simulated using isothermal and isobaric ensemble (NPT) relaxation for 50,000 steps; the atoms were recorded every 100 steps during stretching. Analysis of crystal defects employed Common Neighbor Analysis (CNA) [19]. The deformation mechanism of the polycrystalline Al-Mg-Zn aluminum alloy was monitored and analyzed by dislocation analysis (DXA).

### 2.4. Corrosion Fatigue Crack Propagation

The CFCG test was carried out on a vertical fatigue testing machine (Changchun Qianbang Testing Equipment Co., Ltd., Changchun, China). A 3.5 wt.% NaCl solution was used to simulate the corrosive effect of seawater, and the fatigue crack length was measured by the flexibility method; measurement accuracy was not less than 0.01 mm. The measurement gap opening displacement was calibrated according to the GB/T 12160-2002 [20] standard. The maximum axial loading force was 3.5 KN, the frequency 0.5 Hz, the waveform a sine wave, and the stress ratio = 0.1. According to the GB/T 2012.2-2006 standard [21], the sample was pre-cracked, with the length of the pre-crack being approximately 2 mm. During the test, the number of cycles (N) and the crack length (a) were collected in real time, applying GB/T 6398-2017 [22]. “Fatigue of Metal Materials”, the seven-point incremental polynomial data processing method, was adopted, with the method specified in the “Crack Growth Rate Test Method”. The data were processed by a Visual Basic (VB) programming program (Visual Basic 15.5), where the CFCG behavior can be described by the relationship between the crack growth rate (*da*/*dN*) and the range of the stress intensity (factor *K*).

(1) $$\Delta K=\frac{\Delta P}{B\sqrt{W}}\times \frac{2+\alpha }{(1-\alpha {)}^{3/2}}\times (0.886+4.64\alpha -13.32\alpha^{2}+14.72\alpha^{3}-5.6\alpha^{4})$$

(2) $$\frac{da}{dN}={C(∆K)\;}^{2}$$

In this relationship, *α* = *a*/*W*, where a represents the crack length, *W* is the sample width, *B* is the sample thickness, Δ*P* is the load amplitude, *N* is the number of stress cycles, *C* and m are constants related to the material, and Δ*K* is the stress intensity factor variation range.

## 3. Results and Discussion

### 3.1. Microstructure and Mechanical Properties

**Figure 4:**
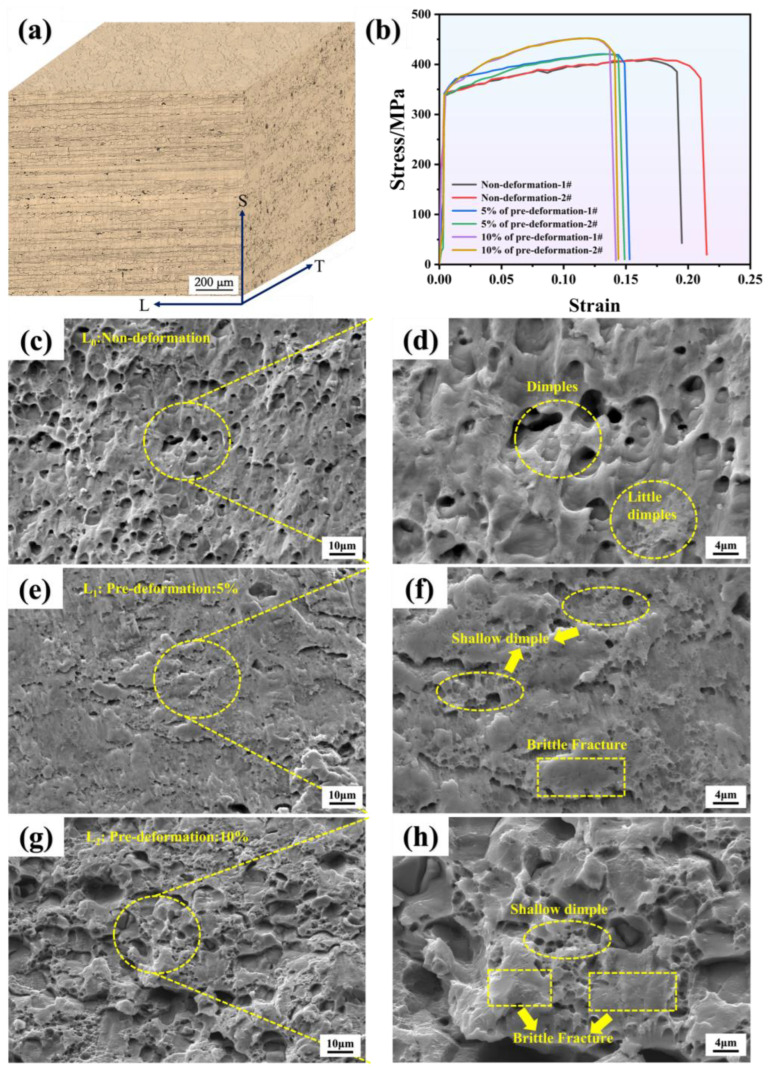
(**a**) Optical microscope image of Al-Mg-Zn alloy: orientation: rolled (L), long transverse (T), and thickness (S). (**b**) Tensile strength of Al-Mg-Zn Alloy after pre-deformation. (**c**,**d**) Tensile fracture of non-deformation. (**e**,**f**) Tensile fracture of 5% of pre-deformation. (**g**,**h**) Tensile fracture of 10% of pre-deformation.

The three-direction microstructure of Al-MgZn Alloy is shown in Figure 4a. The directions are rolling (L), long transverse (T) and thickness (S), respectively. The grains in the L-T plane are uniformly arranged, and pre-deformation is negligible. The grains in the L-S plane are “flattened”, and the grains show obvious fibrous features along the rolling direction.

In addition, the Al matrix is dispersed with fine MgZn_2_ secondary phases. Figure 4b shows the tensile curves of two sets of parallel samples of Al-Mg-Zn alloy under different pre-deformation levels. After pre-deformation, the overall tensile strength increases; the average tensile strength of L_0_ is 410 MPa, L_1_ is 421 MPa, and L_2_ is 451 MPa. It can be seen that, compared to the unstrained condition, the tensile strength increased by 2.63% and 10.00% with pre-deformation of 5% and 10%, respectively. As the pre-deformation level increases, the material’s tensile strength increases. This is because pre-deformation can introduce grain boundaries and dislocations, thereby increasing the material’s dislocation density, enhancing the grain boundary strengthening effect, and improving the material’s tensile strength [23,24].

At the same time, as the pre-deformation level increases, the tensile fracture of the material also exhibits different characteristics. In the unstrained condition, the dimples are deep, indicating good toughness of the material. However, when the pre-deformation level reaches 5% and 10%, the dimples become shallower, and even disappear, with localized areas of smooth surfaces and a certain degree of brittle fracture on the fracture surface. This indicates that the strength of the material increases after pre-deformation, while the plastic toughness decreases. This is bound to have an impact on the fatigue crack propagation of the material in a corrosive environment.

### 3.2. Molecular Dynamics Calculation Results

**Figure 5:**
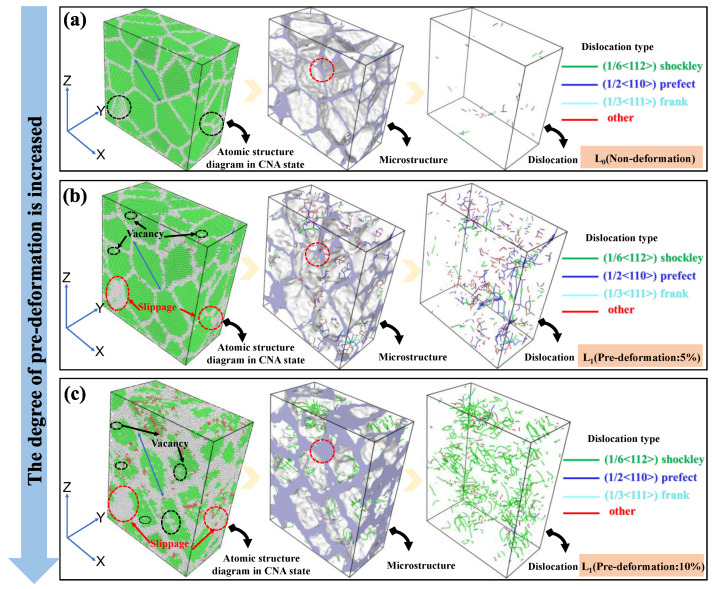
Variation trend of the number of crystal slips, vacancies, and dislocations under different pre-deformation degrees along the crystal direction of [001]: (**a**) Tensile fracture of non-deformation. (**b**) Tensile fracture of 5% of pre-deformation. (**c**) Tensile fracture of 10% of pre-deformation.

As shown in Figure 5, the atomic structure diagram of the stretched [001] crystal direction clearly shows the rotation of the grains and the occurrence of defects such as slip and vacancies in local areas. The greater the pre-deformation, the more pronounced these changes become. Further analysis of the microstructure under different pre-deformation levels reveals that the white disordered atomic regions gradually increase with pre-deformation, leading to an increase in atomic spacing and enhanced atomic disturbance behavior. A large number of disordered atoms in this area release stress, making the proliferation of dislocations relatively easy. By observing the distribution of dislocations, it can be found that as the pre-deformation level increases, dislocations continue to proliferate [25].

Using the Dislocation Extraction Algorithm (DXA) to analyze the internal dislocation situation in different pre-deformation models, the color scheme indicates unknown dislocations in red, 1/2<1 1 0> (perfect) dislocations in blue, 1/6<1 1 2> (Shockley) dislocations in green, and 1/3<1 1 1> (Frank) dislocations in cyan. In the undeformed model, a small number of dislocations appear, mainly dominated by 1/2 (perfect) dislocations. When the pre-deformation reaches 5%, the number of dislocations sharply increases, mainly consisting of 1/2<1 1 0> (perfect) and 1/6<1 1 2> (Shockley) dislocations, with 1/2<1 1 0> (perfect) dislocations playing a dominant role in deformation, while other types of dislocations have relatively smaller line lengths. As the pre-deformation continues to increase to 10%, the number of dislocations continues to increase, with 1/6<1 1 2> (Shockley) dislocations becoming the primary form, playing a dominant role in deformation.

**Figure 6:**
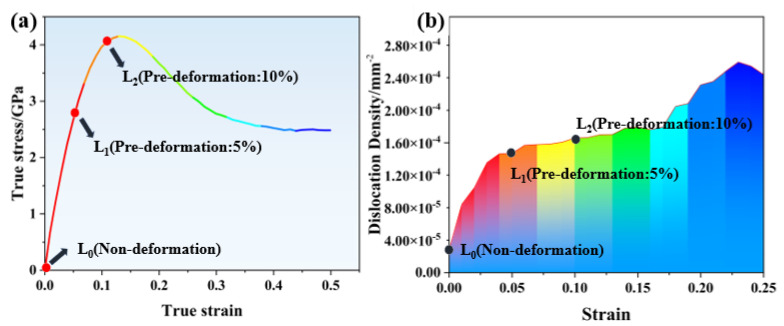
(**a**) Tensile stress–strain curves of Al-Mg-Zn alloy and (**b**) dislocation densities under different degrees of pre-deformation.

Dislocation density is the ratio of dislocation lines to volume [26]. Figure 6 illustrates the relationship between dislocation density and strain under various pre-deformation levels, highlighting the significance of dislocation density, which is defined as the ratio of dislocation lines to volume. As pre-deformation increases, dislocations accumulate, enhancing elastic interactions between them that raise the resistance to dislocation slip and, consequently, the material’s tensile strength, consistent with the tensile test results. However, the non-uniform distribution of dislocations can lead to dislocation pile-ups, causing stress concentration, which may promote the propagation of fatigue cracks. This emphasizes the importance of understanding both the accumulation and distribution of dislocations in optimizing material performance [27,28].

From a comprehensive comparison of dislocation density and type, it can be observed that when pre-deformation reaches a certain level, dislocations accumulate, and the elastic interaction between dislocations increases the resistance to dislocation slip, thereby increasing the material’s tensile strength, consistent with the results of tensile tests [29,30]. However, it is worth noting that the uneven distribution of dislocations can also lead to dislocation pile-ups, causing stress concentration, which to some extent promotes the propagation of fatigue cracks [31].

### 3.3. Corrosion Fatigue Crack Growth Rate Curve

**Figure 7:**
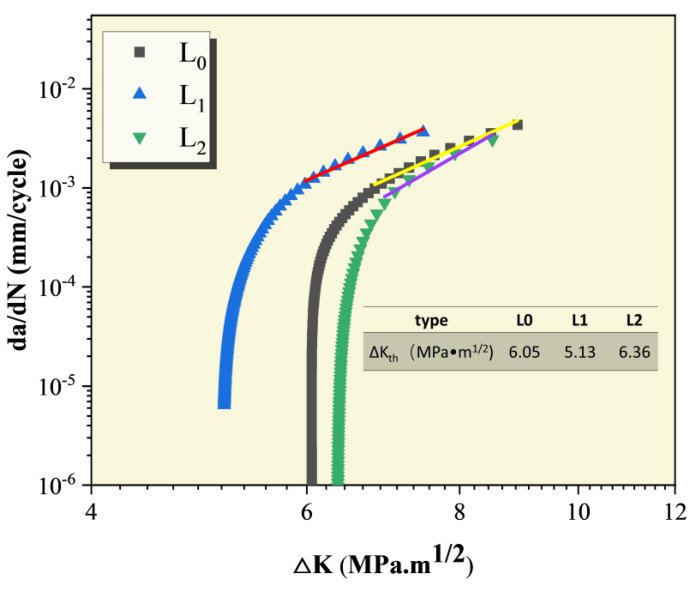
CFCG rate curve of Al-Mg-Zn Alloy after pre-deformation.

Figure 7 shows the crack propagation rate curves of Al-Mg-Zn alloy under different pre-deformation levels in a 3.5 wt.% NaCl solution. Table 2 presents the fitting results of the Paris formula. By comparing the crack propagation rate curves of Al-Mg-Zn alloy under different pre-deformation levels in a 3.5 wt.% NaCl solution environment, it can be observed that the difference in pre-deformation levels leads to differences in crack propagation threshold values. The crack propagation threshold values under three pre-deformation states are in the following order from highest to lowest: L2 (6.36 MPa·m^1/2^) > L0 (6.05 MPa·m^1/2^) > L1 (5.13 MPa·m^1/2^). A lower threshold value indicates easier crack propagation. This is attributed to the change in the internal structure and microstructure of the material due to pre-deformation under external stress. The crack propagation threshold value is lowest for 5% pre-deformation, resulting in the fastest crack propagation rate. The threshold value is highest for 10% pre-deformation, leading to the lowest propagation rate, while the threshold value and propagation rate for no pre-deformation tend to be in between.

The reason for these results is that pre-deformation increases the density of internal dislocations in the material, leading to enhanced grain boundary strengthening effects and an increase in material strength, which to some extent inhibits crack propagation. However, pre-deformation may also cause dislocation pile-ups, resulting in significant stress concentration, making crack propagation easier in regions with higher stress under fatigue loading. Combining the results of tensile tests and the evolution of dislocations during pre-deformation through molecular dynamics simulations, it can be concluded that the distribution of dislocations is non-uniform under 5% pre-deformation, leading to stress concentration, while the density of dislocations is increased under 10% pre-deformation, but the distribution is more uniform, increasing the material strength and also raising the threshold value for crack propagation, hindering crack propagation. The threshold value for no pre-deformation lies between the two. This indicates that while pre-deformation enhances the material’s strength, it also leads to a decrease in fatigue performance, highlighting the critical importance of selecting an appropriate pre-deformation level.

### 3.4. Corrosion Fatigue Crack Propagation Path

**Figure 8:**
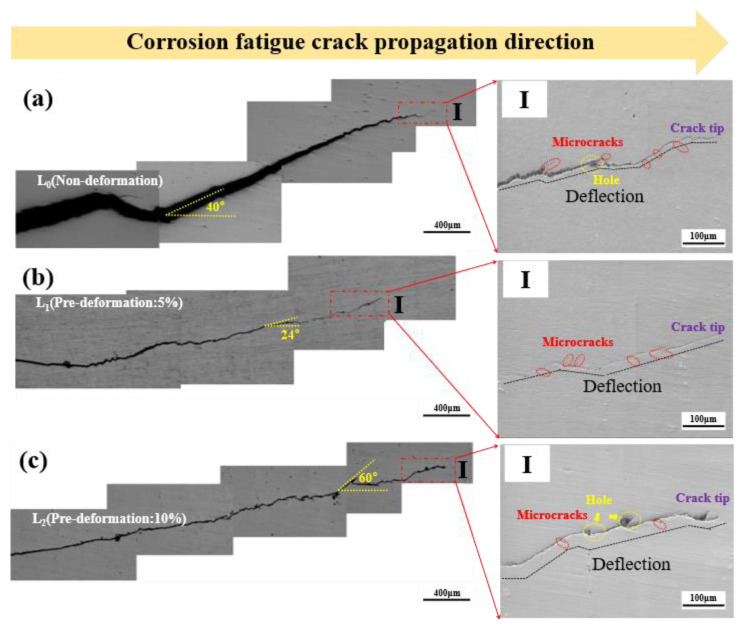
Corrosion fatigue crack propagation path of Al-Mg-Zn Alloy with different degrees of pre-deformation: (**a**) non-deformation L_0_, (**b**) pre-deformation L_1_, and (**c**) pre-deformation L_2_.

To further clarify the differences in the CFCG rates, an analysis of the characteristics of the crack propagation paths was conducted. Figure 8 displays macroscopic photographs of the cracks in Al-Mg-Zn alloy under different pre-deformation levels. As shown in Figure 8a, the crack under no pre-deformation exhibits a typical “Z”-shaped path, with an angle of approximately 40° with the horizontal direction. Additionally, there are micro-cracks parallel or perpendicular to the main crack direction. The crack path under 5% pre-deformation (Figure 8b) is relatively straight, with the crack propagation direction at an angle of around 24° with the horizontal direction. Compared to no pre-deformation, there are more secondary cracks at certain angles to the main crack. The crack path under 10% pre-deformation (Figure 8c) is more similar to the unstrained condition (Figure 8a), with the crack propagation direction at an angle of approximately 60° with the horizontal direction. The results indicate significant differences in the crack propagation paths under different pre-deformation levels. The crack path under 5% pre-deformation is straighter, smoother, and has the smallest angle with the horizontal direction, with the highest number of secondary cracks. This suggests that the embrittlement of the crack tip region is more pronounced at 5% pre-deformation, resulting in a lower crack propagation threshold value and a faster propagation rate, corresponding to the crack propagation rate curve (Figure 7).

**Figure 9:**
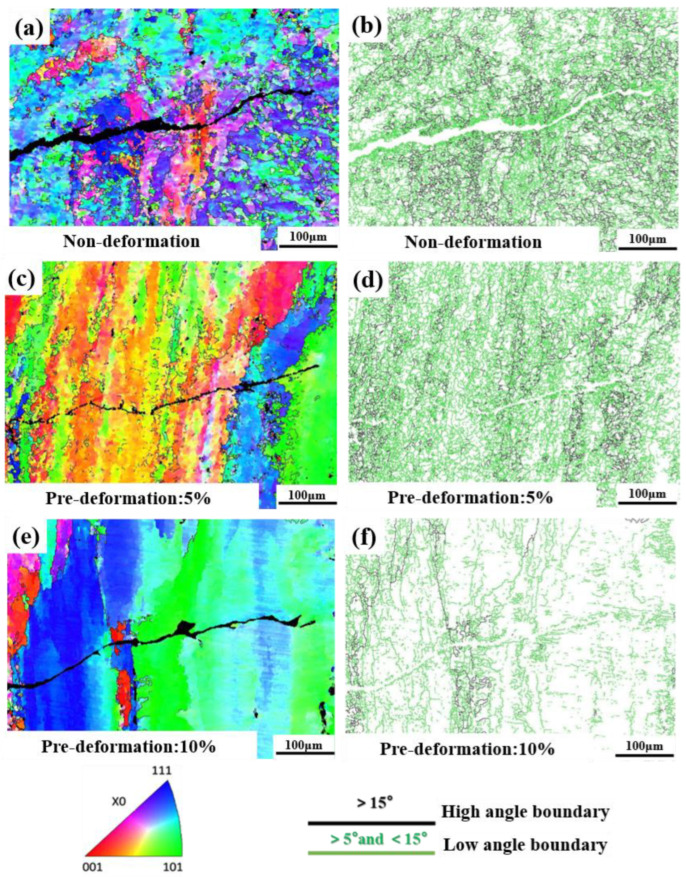
EBSD images of Al-Mg-Zn Alloy with different degrees of pre-deformation: (**a**) IPF maps of non-deformation, (**b**) grain boundary of non-deformation, (**c**) IPF maps of pre-deformation: 5%, (**d**) grain boundary of pre-deformation: 5%, (**e**) IPF maps of pre-deformation: 10%, and (**f**) grain boundary of pre-deformation: 10%.

Using scanning electron microscopy combined with EBSD, further analysis of the crack tip CFCG path and microstructural changes was conducted (Figure 9). The IPF maps of the crack tips of the non-deformation sample L0 and pre-deformation samples L1 (5%) and L2 (10%) are shown in Figure 9a,c,e, respectively. The analysis indicates that the corrosion fatigue cracks in all three conditions exhibit a mixed mode of transgranular and intergranular crack propagation during the expansion process, with transgranular propagation being predominant. The distribution of grain boundaries at the crack tip is shown in Figure 9b,d,f, where high-angle grain boundaries are represented in black and low-angle grain boundaries are represented in green. With the increase in pre-deformation level, the number of high-angle grain boundaries decreases. This is due to the deformation and slip of grain boundaries caused by plastic deformation, leading to changes in the shape and position of the grain boundaries [23]. Additionally, a large number of subgrains form, and due to the small orientation differences between adjacent subgrains, it is difficult to distinguish them by color. Furthermore, in the strain region, the deformation reduces the orientation differences of the grain boundaries, making the grain boundaries less distinct. From the perspective of crack morphology, there is significant crack deflection in the crack path with 10% pre-deformation, which to some extent impedes the crack propagation.

**Figure 10:**
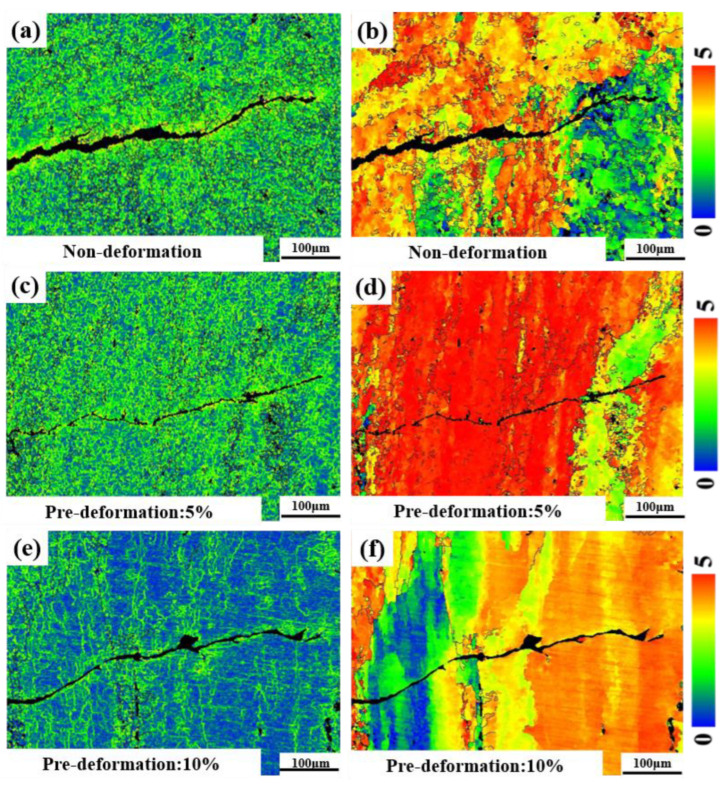
EBSD images of Al-Mg-Zn Alloy with different degrees of pre-deformation: (**a**) KAM maps of non-deformation, (**b**) Schmid factor map of non-deformation, (**c**) KAM maps of pre-deformation: 5%, (**d**) Schmid factor map of pre-deformation: 5%, (**e**) KAM maps of pre-deformation:10%, and (**f**) Schmid factor map of pre-deformation: 10%.

The Kernel Average Misorientation (KAM) images (Figure 10a,c,e) represent the degree of plastic deformation at the crack tip, with green indicating irregular residual strain. It can be seen that the distribution of residual strain near the crack tip is consistent with the distribution of small-angle grain boundaries. During the process of corrosion fatigue crack propagation, significant plastic deformation occurs near the crack. Compared to no deformation and 5% pre-deformation, the residual strain at the crack tip is smaller and the plastic deformation is more uniform with 10% pre-deformation. The Schmid factor represents the ease of activation of slip systems and is used to evaluate the plastic deformation ability of single-crystal grains. The Schmid factor map at the crack tip position is shown in Figure 10b,d,f. A larger Schmid factor indicates a soft orientation in the material structure, which is conducive to crack propagation. When the Schmid factor is smaller, it is difficult for the material to undergo slip, indicating a hard orientation in the structure. The Schmid factor is larger when the pre-deformation is 5%, leading to a faster crack propagation rate, which is consistent with the distribution trend of the crack propagation threshold and crack propagation rate [32,33].

**Figure 11:**
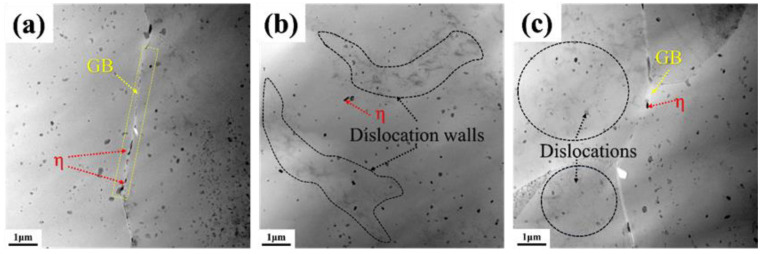
TEM images of the corrosion fatigue near fracture zone of Al-Mg-Zn alloy with different deformation: (**a**) no deformation; (**b**) 5% pre-deformation; and (**c**) 10% pre-deformation.

Figure 11 shows TEM images of the near-fracture region of Al-Mg-Zn alloy with different pre-deformation during corrosion fatigue. As shown in Figure 11a, the alloy under no deformation has a low dislocation density, with a large amount of MgZn_2_ (η phase) distributed at grain boundaries and within the grains. From Figure 11b, it can be observed that when the pre-deformation is 5%, the dislocation density of the alloy significantly increases, and the distribution of dislocations becomes uneven [34]. Local accumulation of dislocations forms dislocation walls in the matrix, surrounded by the η phase, creating stress concentration areas and increasing the risk of cracking. In Figure 11c, when the pre-deformation increases to 10%, although the dislocation density continues to increase, the distribution of dislocations becomes more uniform, to some extent reducing the stress concentration and improving the material’s deformation ability [35]. This is consistent with the trend in dislocation distribution during tensile deformation in molecular dynamics simulations.

### 3.5. Fractography

**Figure 12:**
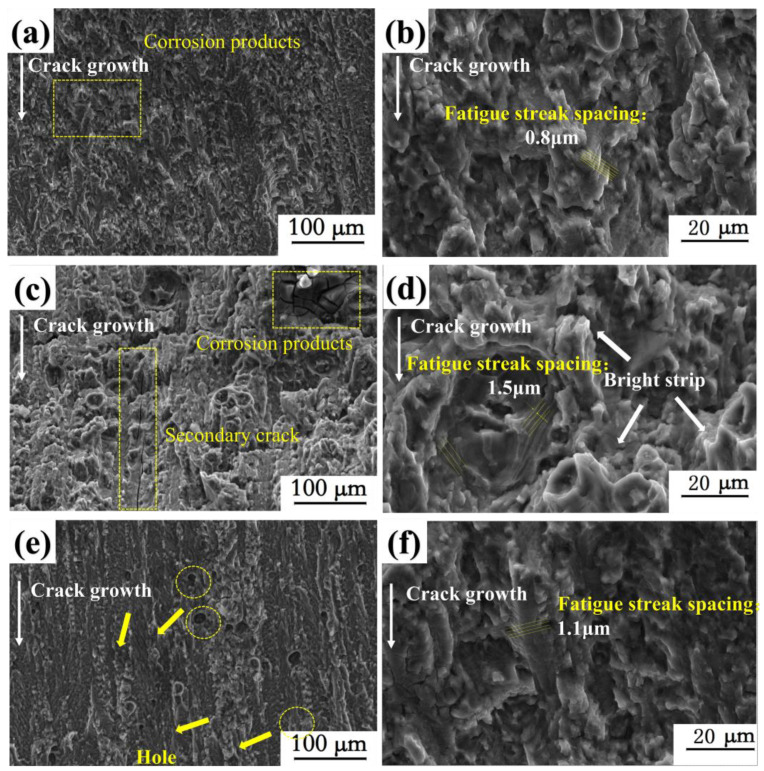
Micromorphology of corrosion fatigue crack propagation fracture of samples with different degrees of pre-deformation in 3.5 wt.% NaCl solution. (**a**,**b**) L_0_: non-deformation; (**c**,**d**) L_1_: 5% pre-deformation; and (**e**,**f**) L_2_: 10% pre-deformation.

Figure 12 shows the micro-fractures associated with the CFCG of Al-Mg-Zn alloy under different degrees of pre-deformation. It can be seen that the fracture morphologies are quite distinct. Compared with no deformation L0 (Figure 12a,b), the specimens after pre-deformation, L_1_ (Figure 12c,d) and L_2_ (Figure 12e,f), exhibit a relatively flat corrosion fatigue fracture. We can attribute this response to the increase in the strength of the pre-deformation samples, the decrease in plasticity, and the alteration to the material structure. Moreover, a large number of holes left by the detachment of the second phase can be seen on the fracture, with many secondary cracks along the direction of fatigue crack propagation for pre-deformation: 5%. Clear secondary cracks can be seen in the fracture with 5% pre-deformation, which will lead to local stress concentration at the crack tip and accelerate the process of crack propagation, which is consistent with the trend in the threshold value and rate of crack propagation. Higher-magnification analysis allowed a more detailed consideration of fracture morphology. Fatigue striations are relatively blurred, but striations with different spacings can still be seen locally. The spacing for sample L_0_ is about 0.8 μm, whereas that for L_1_ and L_2_ is, respectively, 1.5 μm and 1.1 μm. The fatigue striation spacing reflects the force crack growth rate to a certain extent, and the spacing for L1 is the largest. In addition, the crack growth rate test has also shown that the crack growth rate of L1 is highest. Undulating bright bands are a feature of L_1_ SEM images, which may be caused by damage to the crack tip. This indicates that a certain degree of pre-deformation can enhance the anti-corrosion response, but excessive pre-deformation inhibits this effect in terms of fatigue performance [36].

### 3.6. Mechanism Analysis

The pre-deformation of Al-Mg-Zn alloy affects its corrosion fatigue crack propagation behavior. The microstructure of Al-Mg-Zn alloy after pre-deformation changes due to the deformation of the material. The aluminum alloy structure is more chaotic and grain deformation is significant, hampering the resistance of the material to crack growth. Such pre-deformation will lead to a reduction in the corrosion fatigue crack growth threshold for Al-Mg-Zn alloy. Greater pre-deformation results in work hardening, which serves to increase material tensile strength, which is manifested as an increase in the CFCG threshold and a decrease in the crack growth rate [37]. Tibor Berecz [38] studied the pre-deformation behavior of 6082 aluminum alloy and found that the dislocation density increased significantly with an increase in the degree of pre-deformation deformation. The elastic interaction increases the resistance to dislocation sliding, which eventually leads to a significant increase in tensile strength, so the CFCG threshold of 10% pre-deformation increases. Wang [39] studied the microstructure and tensile properties of industrially prepared 7050 aluminum alloy plates. Their results showed that the grain size distribution in the aluminum alloy was not uniform, and some grains exhibited coarseness, which can lead to uneven deformation during the pre-deformation process. Chen [40] studied the torsional thermal deformation behavior of 7A04 aluminum alloy. They reported differences in grain shape in different regions (equiaxed, elliptical, and fibrillar) due to deformation, resulting in different properties with pre-deformation. This can also be seen in our 3D metallographic analysis, given in Figure 4a. Yang [41] examined the effect of different strain rates on recrystallization and flow stress in 7085 aluminum alloy during hot compression, and found that high strain rates promote the formation of a grain boundary structures. Jian-Liang [42] used the K-M dislocation density model to quantitatively assess the dislocation density of 7A85 aluminum alloy during hot deformation. The parameters in the model were determined by the stress–true strain curve obtained from hot compression experiments. Their two-stage K-M dislocation density model [42,43,44,45] considered that some dislocations transformed into dislocation cells or sub-crystals, which retained a high dislocation density. Their work established that an increase in plasticity was accompanied by an increased dislocation density, but the dislocation density inside the original crystal was ignored.

**Figure 13:**
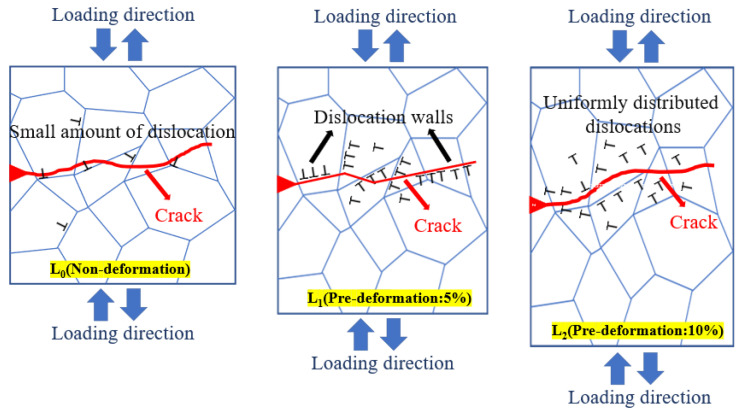
Crack propagation paths under different degrees of dislocation density.

Based on the current research results, the influence of pre-deformation on the mechanism of corrosion fatigue crack propagation has not been studied. We conducted an in-depth analysis of the tensile strength and fracture surfaces of Al-Mg-Zn alloy under different pre-deformation levels, and elucidated the role of pre-deformation in the fracture mechanism. Additionally, molecular dynamics simulations were used to analyze the formation process of defects such as dislocations and vacancies under different pre-deformation levels, and the variation in dislocation density under pre-deformation conditions. Based on these research results, corrosion fatigue crack propagation tests were conducted to obtain the crack propagation threshold and rate under different pre-deformation levels. Combined with characterization methods such as EBSD, SEM, and TEM, the characteristics along the crack propagation path, fracture morphology, and the variation of dislocation density near the crack tip were analyzed. As shown in Figure 13, compared to the experiments with non-deformation and 10% pre-deformation, the threshold value is lowest and the crack propagation rate is fastest with 5% pre-deformation. This is mainly due to the increase in dislocation density and material strength caused by pre-deformation. However, under 5% pre-deformation, the accumulation of dislocations forms dislocation walls, resulting in significant stress concentration, making it easier for the crack to propagate along the stress concentration areas. On the other hand, under 10% pre-deformation, the distribution of dislocations is more uneven, increasing the material strength while also increasing the resistance to crack propagation [46,47].

## 4. Conclusions

This study examines the impact of pre-deformation on the corrosion fatigue crack propagation (CFCG) of Al-Mg-Zn alloy in a corrosive environment. Through tensile tests and molecular dynamics simulations, we assessed how varying pre-deformation levels affected dislocation density and ultimately influenced the alloy’s mechanical properties and crack propagation behavior in a 3.5% NaCl solution.

1. Enhanced Tensile Strength: The results show that pre-deformation improves the tensile strength of Al-Mg-Zn alloy, with increases of 2.63% and 10.00% at 5% and 10% pre-deformation, respectively, compared to the unstrained condition.
2. Influence on Crack Propagation: Corrosion fatigue crack propagation is significantly affected by pre-deformation, with thresholds varying as L2 (6.36 MPa·m^1/2^) > L0 (6.05 MPa·m^1/2^) > L1 (5.13 MPa·m^1/2^). Higher dislocation densities at 10% pre-deformation enhance resistance to crack propagation.
3. Molecular dynamics simulations showed that as the pre-deformation level increased, the dislocation density also increased, consistent with the results of the tensile tests. However, the uneven distribution of dislocations also led to stress concentration, which to some extent promoted the propagation of corrosion fatigue cracks.

## Figures and Tables

**Table 1:** Al-Mg-Zn alloy composition (wt.%).

Material	Zn	Mg	Cu	Mn	Si	Ti	Cr	Fe	Al
Al-Mg-Zn alloy	4.0~5.0	1.0~1.8	0.10	0.20~0.70	0.35	0.01~0.06	0.06~0.20	0.40	Bal.

**Table 2:** Paris formula fitting results.

Degree of Pre-Deformation	Constant C	Constant m	Paris Formula
L_0_ (non-deformation)	7.09 × 10^−8^	5.43	*da*/*dN* = 7.09 × 10^−8^(Δ*K*)^5.43^
L_1_ (5% pre-deformation)	2.66 × 10^−8^	5.23	*da*/*dN* = 2.66 × 10^−8^(Δ*K*)^5.23^
L_2_ (10% pre-deformation)	8.41 × 10^−10^	7.11	*da*/*dN* = 8.41 × 10^−10^(Δ*K*)^7.11^

## Data Availability

The original contributions presented in this study are included in the article. Further inquiries can be directed to the corresponding authors.
